# Micro Regional Heterogeneity of ^64^Cu-ATSM and ^18^F-FDG Uptake in Canine Soft Tissue Sarcomas: Relation to Cell Proliferation, Hypoxia and Glycolysis

**DOI:** 10.1371/journal.pone.0141379

**Published:** 2015-10-26

**Authors:** Kamilla Westarp Zornhagen, Anders E. Hansen, Jytte Oxboel, Andreas E. Clemmensen, Henrik H. El Ali, Annemarie T. Kristensen, Andreas Kjær

**Affiliations:** 1 Department of Veterinary Clinical and Animal Sciences, Faculty of Health and Medical Sciences, University of Copenhagen, Frederiksberg C, Denmark; 2 Department of Clinical Physiology, Nuclear Medicine & PET and Cluster for Molecular Imaging, Rigshospitalet and University of Copenhagen, Copenhagen N, Denmark; 3 Department of Micro- and Nanotechnology, Technical University of Denmark, Kgs. Lyngby, Denmark; The University of Chicago, UNITED STATES

## Abstract

**Objectives:**

Tumour microenvironment heterogeneity is believed to play a key role in cancer progression and therapy resistance. However, little is known about micro regional distribution of hypoxia, glycolysis and proliferation in spontaneous solid tumours. The overall aim was simultaneous investigation of micro regional heterogeneity of ^64^Cu-ATSM (hypoxia) and ^18^F-FDG (glycolysis) uptake and correlation to endogenous markers of hypoxia, glycolysis, proliferation and angiogenesis to better therapeutically target aggressive tumour regions and prognosticate outcome.

**Methods:**

Exploiting the different half-lives of ^64^Cu-ATSM (13h) and ^18^F-FDG (2h) enabled simultaneous investigation of micro regional distribution of hypoxia and glycolysis in 145 tumour pieces from four spontaneous canine soft tissue sarcomas. Pairwise measurements of radioactivity and gene expression of endogenous markers of hypoxia (HIF-1α, CAIX), glycolysis (HK2, GLUT1 and GLUT3), proliferation (Ki-67) and angiogenesis (VEGFA and TF) were performed. Dual tracer autoradiography was compared with Ki-67 immunohistochemistry.

**Results:**

Micro regional heterogeneity in hypoxia and glycolysis within and between tumour sections of each tumour piece was observed. The spatial distribution of ^64^Cu-ATSM and ^18^F-FDG was rather similar within each tumour section as reflected in moderate positive significant correlations between the two tracers (*ρ* = 0.3920–0.7807; p = 0.0180 –<0.0001) based on pixel-to-pixel comparisons of autoradiographies and gamma counting of tumour pieces. ^64^Cu-ATSM and ^18^F-FDG correlated positively with gene expression of GLUT1 and GLUT3, but negatively with HIF-1α and CAIX. Significant positive correlations were seen between Ki-67 gene expression and ^64^Cu-ATSM (*ρ* = 0.5578, p = 0.0004) and ^18^F-FDG (*ρ* = 0.4629–0.7001, p = 0.0001–0.0151). Ki-67 gene expression more consistently correlated with ^18^F-FDG than with ^64^Cu-ATSM.

**Conclusions:**

Micro regional heterogeneity of hypoxia and glycolysis was documented in spontaneous canine soft tissue sarcomas. ^64^Cu-ATSM and ^18^F-FDG uptakes and distributions showed significant moderate correlations at the micro regional level indicating overlapping, yet different information from the tracers.^18^F-FDG better reflected cell proliferation as measured by Ki-67 gene expression than ^64^Cu-ATSM.

## Introduction

Phenotypic and cellular heterogeneity within malignant tumours has been recognized since the early days of cancer biology [1–3]. All constituents of the malignant tumour contribute to its heterogeneity. Even though cancers were initially seen as solely consisting of cancer cells, they are now appreciated as complex structures comprising also stromal cells and non-cellular constituents [4–6]. Thus complex mechanisms involving both the cancer cells, with their acquired hallmarks [7, 8], and the tumour microenvironment appear important during carcinogenesis, progression and metastasis [8–10] and simultaneously contribute to the cellular and phenotypic heterogeneity of malignant tumours [3, 11].

Key factors in the microenvironment of malignant tumours include hypoxia, glycolysis and proliferation. Cancer cells cover their energy demands through aerobic glycolysis (Warburg effect), which also supplies the necessary building blocks for the biomass formed during continuous cancer cell proliferation [12–14]. Rapidly growing cancer cells will experience diffusion-limited hypoxia as soon as the intercapillary distance of 120–130 μm for oxygen delivery is exceeded [15, 16]. Hypoxia, through activation of hypoxia inducible factors (HIF), especially HIF-1α, regulates a myriad of genes leading to adaptive and genetic changes in the cancer cells resulting in survival advantages [17]. The changes induced by hypoxia include induction of angiogenesis, increased glycolytic activity and increased proliferation. As the newly formed tumour vasculature is highly disorganized and dysfunctional, impaired blood flow and consequently perfusion-limited hypoxia will result [15, 18]. Furthermore waste products from increased glycolysis, such as lactate will acidify the tumour microenvironment necessitating further adaptations [14]. Thus the complex interplay and the changes occurring in the tumour microenvironment result in a vicious circle driving the cancer cells towards a more aggressive and malignant phenotype showing treatment resistance [17, 19–24]. As hypoxia, increased glycolytic activity and proliferation have been associated with a poor prognosis in different human cancers [25–31], it is of special interest to evaluate cancers and potentially target therapy with respect to these microenvironmental phenotypes and their heterogeneity.

Insights into the molecular aspects of the tumour microenvironment can be accomplished non-invasively via positron emission tomography (PET). 2-deoxy-2-[^18^F]fluoro-D-glucose (^18^F-FDG) is routinely used to detect malignant tumours due to their high glycolytic activity. Furthermore it has been proposed as a surrogate marker of tumour hypoxia [32] due to the close link between hypoxia and increased glycolytic activity. However, studies comparing ^18^F-FDG uptake with different hypoxia PET tracers have been inconsistent and have shown regional and tumour type differences in distribution of hypoxia and glycolysis [33–36].

One promising hypoxia selective PET tracer is Cu-diacetyl-bis(N^4^-methylthiosemicarbazone) (Cu-ATSM) [37], which labelled with different copper radioisotopes, has been shown to reflect hypoxia in human studies of cervical cancer [27, 38]. Only a few comparative PET studies of ^64^Cu-ATSM and ^18^F-FDG have been performed in canine and human cancer patients showing from moderate or strong positive significant correlations [39] to no significant correlations [40]. However, due to the detection method in PET, simultaneous investigation of two tracers is not possible. Dual tracer PET studies are thus subject to a time difference between the scans during which changes in the microenvironment may occur. Furthermore the spatial resolution of PET is limited considering the microscopic distances under which changes in the microenvironment may occur and thus the true micro regional heterogeneity may not be registered. Simultaneous dual tracer comparisons of ^64^Cu-ATSM and ^18^F-FDG at the micro regional level using autoradiography have only been performed in preclinical rodent tumour studies showing poor correlations [41–45]. Recently a study described a comparison between the micro regional extent of hypoxia measured by the radioactivity of ^18^F-fluoroazomycin arabinoside (FAZA) with gene expression of endogenous markers of hypoxia in small tumour fragments [46]. A technique for pairwise measurement of mRNA transcript levels and hypoxia examined by radioactivity of FAZA in small tumour fragments was described in mice with murine and xenografted human tumours. The study concluded that carbonic anhydrase IX and glucose transporter 1 were strongly correlated to hypoxia meassured by FAZA [46].

However, little is still known about the in vivo micro regional heterogeneity of the tumour microenvironment regarding hypoxia, glycolysis and proliferation in spontaneous solid tumours, as it is difficult to obtain adequate and representative tumour samples from human cancer patients. As stated earlier, knowledge about the micro regional heterogeneity of the tumour microenvironment may be of importance for the prognosis of a cancer patient and furthermore could pave the way for more targeted and individualized treatment approaches. Therefore the aim was to simultaneously investigate the micro regional heterogeneity of hypoxia and glycolysis in canine cancer patients with spontaneous soft tissue sarcomas, examining the uptake and distribution of ^64^Cu-ATSM and ^18^F-FDG, their potential spatial overlap as well as their correlation to endogenous markers of hypoxia (HIF-1α, carbonic anhydrase IX (CAIX)), glycolysis (hexokinase 2 (HK2) and glucose transporters GLUT1 and GLUT3), proliferation (Ki-67) and angiogenesis (vascular endothelial growth factor (VEGFA) and tissue factor (TF)). The canine cancer model allowed for these studies to be performed in “full-size” compared to human patients. It was hypothesized that micro regional heterogeneity would be observed; that ^64^Cu-ATSM and ^18^F-FDG would show varying degrees of overlap and correlation; that ^64^Cu-ATSM would correlate positively with gene expression of endogenous markers of hypoxia, glycolysis and angiogenesis due to the important function of HIF-1α in their regulation; that ^18^F-FDG would correlate with gene expression of genes involved in glycolysis; and lastly that ^64^Cu-ATSM and ^18^F-FDG also to some extent would reflect tumour proliferation.

## Materials and Methods

### Ethics statement

The study protocol was approved by the Animal Ethics and Administrative Committee at the Department of Veterinary Clinical and Animal Sciences, Faculty of Health and Medical Sciences, University of Copenhagen, Denmark. Owners provided written informed consent prior to inclusion of their dog.

### Study population

Canine cancer patients with histologically confirmed soft tissue sarcoma admitted to the University Hospital for Companion Animals at the Department of Veterinary Clinical and Animal Sciences, Faculty of Health and Medical Sciences, University of Copenhagen Denmark from 2011 to 2013 were eligible for inclusion, if curable intent surgery was not possible or the owner declined advanced surgery. Treatment consisting of tumour debulking followed by radiotherapy was offered through participation in the study. Four canine cancer patients were prospectively enrolled in the study (Table 1). As the study focus was on micro regional heterogeneity within each individual tumour, the number of tumour pieces from each individual tumour was of greater interest than the number of patients included. The canine cancer patients and their owners had to fulfil a number of presuppositions to be eligible to the research project (transportation between research localities, owner agreement, individual animal cooperation), thus far from all cancer patients with soft tissue sarcomas admitted to the University Hospital for Companion Animals could be included. To eliminate potential variations in results attributable to inclusion of several cancer types, only spontanous canine soft tissue sarcomas were eligible for inclusion.

**Table 1:** Patient and tumour characteristics for the four canine cancer patients included in the study.

Patient no.	Tumour type	Location	Grade	Size	Age of dog at surgery
1	Recurrent soft tissue sarcoma	Lateral carpus	2	22mm x 14,8mm x 1cm	8 yr
2	Soft tissue sarcoma (most likely peripheral nerve sheath tumour or a perivascular wall tumour)	Lateral cheek	1	5,26cm x 7,2cm	10.5 yr
3	Recurrent soft tissue sarcoma (features of perivascular wall tumour)	Lateral thigh	1	3cm x 2cm	8 yr
4	Maxillary fibrosarcoma	Dorso-lateral nose	1	Approximately 7cm x 7cm	6 yr

*Yr* years.

Due to missing data and too high reference gene instabilities (see below) patient 1 was included only in the final analysis of the correlation between ^64^Cu-ATSM and ^18^F-FDG uptake based on gamma counting.

### Experimental setup

The canine cancer patients had a full oncological workup including staging. Prior to planned surgery, the patients were fasted for 12 hours and blood samples confirmed normal serum glucose concentrations and readiness for anaesthesia. Approximately 3 hours prior to surgery ^64^Cu-ATSM (Hevesy Laboratory, DTU Campus Risø, Denmark) was injected intravenously as a bolus. ^64^Cu-ATSM activity in the syringe was recorded and the decay corrected injected activity calculated. The mean received dose of ^64^Cu-ATSM activity was 8.2 MBq/kg (range 7.2–9.5 MBq/kg). Approximately 1 hour prior to surgery the patients received an intravenous bolus of ^18^F-FDG (Department of Clinical Physiology, Nuclear Medicine and PET, Rigshospitalet, Denmark) with a mean activity of 7.8 MBq/kg (range 7.3–8.0 MBq/kg). All patients were monitored after tracer administration. By utilizing the different half-lives of these two tracers it was possible to investigate the two molecular targets (hypoxia and glycolysis) simultaneously. Patients were pre-medicated, anesthetized and monitored during surgery according to the standards of the University Hospital for Companion Animals (pre-medication with Methadone 0.2–0.3 mg/kg IM/IV, Diazepam 0.3mg/kg IM/IV or Ketamine and Midazolam, and if necessary Atropine 0.02mg/kg IM; anaesthesia induction with Propofol 4mg/kg IV and maintenance through Sevoflurane or Isoflurane gas anaesthesia). All patients received post-operative analgesia, and three patients (no. 1, 3 and 4) received post-operative radiotherapy of 6.5–7 Gy once a week for 4 weeks.

As soon as a tumour was excised, it was cut into variable sized pieces for either a) autoradiography (AR) and immunohistochemistry (IHC) (2–4 pieces per tumour) or b) gamma counting and quantitative real-time polymerase chain reaction (qPCR) (30–45 pieces per tumour). A total of 145 tumour pieces from the four soft tissue sarcomas were examined.

### Dual tracer autoradiography and image analysis

Tumour pieces for AR and IHC (approximately 10x10x10 mm) were snap-frozen in liquid nitrogen and transferred to dry ice. Tumour pieces were embedded with Jung Tissue freezing Medium® (Leica Microsystems A/S, Ballerup, Denmark), cut into 8μm tissue sections on a cryostat (Leica CM1850, Leica Microsystems A/S, Ballerup, Denmark) and thaw mounted on microscope slides (Thermo Scientific Menzel-Gläser Superfrost Ultra Plus® microscope slides, Gerhard Menzel GmbH, Braunschweig, Germany). Ten to twenty cryostat sections 215 μm apart were made for each tumour. Slides with tissue sections were exposed to a phosphor imaging screen (MultiSensitive Storage Screen, PerkinElmer, Waltham, MA, USA) for approximately 2 hours. The exposed imaging screens were scanned at 600 dpi (pixel size 42 μm) using a phosphor imaging system (Cyclone® Plus, Storage Phosphor System, PerkinElmer, Waltham, MA, USA) to obtain the first image (^18^F-FDG + ^64^Cu-ATSM). After approximately 12 hours for residual ^18^F-FDG to decay, slides were re-exposed to the phosphor imaging screen for approximately 11 hours before the second reading. This was to ensure that the second scan strictly expressed ^64^Cu-ATSM. All exposures were conducted at 4°C and slides subsequently frozen and stored at -80°C until IHC analysis.

All AR images (pixel size 42 μm) were exported from OptiQuant™ Image Analysis Software (PerkinElmer, Waltham, MA, USA) in TIFF format and transferred for further analysis using in-house developed software in MATLAB (Version R2014a, The Mathworks, Inc., Natick, MA, USA). The AR images of individual tumour tissue sections were cropped from a large image of the whole phosphor imaging screen containing multiple sections. The two individual tumour images (combined ^18^F-FDG + ^64^Cu-ATSM and pure ^64^Cu-ATSM exposure) were co-registered using a rigid transformation and built-in functions, and their intensity corrected linearly for exposure time and scan time difference. By subtracting the pure ^64^Cu-ATSM from the combined image, an estimated pure ^18^F-FDG exposure was obtained. After quadruple down sampling to reduce registration artefacts, direct pixel-to-pixel correlation of the two radioisotopes was made and plotted in a scatter plot. Two Gaussian distributions were fitted to the data using hierarchical cluster analysis, one for image background and one for the AR slide itself, and linear fits and coefficients of determination (R^2^) were calculated for each distribution.

### Ki-67 immunohistochemistry and comparison with autoradiography

Four to eight cryostat sections (most often 430 μm apart) from each canine cancer patient previously used for AR were thawed, followed by fixation in acetone at 4◦C for 10 min. Manual staining procedures were performed as follows: After air-drying, antigen retrieval with microwave heating in citrate buffer, followed by a rinse in phosphate buffered saline (PBS) with 0.1% Tween, was performed. Endogenous enzyme activity was blocked through 8 min soak with Dako REAL^TM^ peroxidase-blocking solution (Dako, Glostrup, Denmark) and 10 min soak with 2% bovine serum albumin (BSA). Incubation with monoclonal mouse anti-human antibody for Ki-67 antigen clone MIB-1 (Dako, Glostrup, Denmark) lasted 1 hour at room temperature at a concentration of 1:200. This antibody also cross-reacts with canine Ki-67. The secondary antibody, an anti-mouse peroxidase labelled polymer (Envision+ System-HRP, Dako, Glostrup, Denmark) was linked to the primary antibody during 40 min of incubation and peroxidase activity was visualized through addition of DAB_+_ Subtrate Chromogen (Dako, Glostrup, Denmark) as detection system. Counterstaining was performed with Mayer’s haematoxylin (Clinical Pharmaceutical Service, Copenhagen University Hospital Pharmacy, Denmark) before dehydration and mounting of cover glass. Each of the described steps until and including DAB_+_ was followed by rinses with PBS, except between the BSA and primary antibody. An 8 μm cryostat section of canine epidermis was included in the run as a positive control of staining only in the basal layer. The rest of the epidermis simultaneously served as negative control tissue. Furthermore a negative control, where the primary antibody was replaced with nonsense anti-body (FLEX Negative Control Mouse, Dako, Glostrup, Denmark) was also included.

IHC slides were scanned with Axio Scan.Z1slide scanner (Carl Zeiss Microscopy GmbH, Jena, Germany) and images (pixel size 0.22 μm) processed in ZEN 2012 software (Carl Zeiss Microscopy GmbH, Jena, Germany). Images resized to 25% (pixel size 0.88 μm) of the original were exported from ZEN 2012 as PNG files for subsequent analysis of correlation between the Ki-67 IHC and ^64^Cu-ATSM and ^18^F-FDG AR images. IHC images (one per tumour) were processed and manually co-registered with AR images of ^64^Cu-ATSM and ^18^F-FDG using ImageJ (public domain, Java-based image processing program) and MATLAB (version R2014b, The Mathworks, Inc., Natick, MA, USA). Colour de-convolution of the IHC images focussing on the brown colour indicating Ki-67 staining and the blue colour indicating nuclear staining was performed. The original IHC image rescaled to a pixel size of 42 μm (size of AR) using pixel averaging with bicubic interprolation was used for manual co-registration with AR images. A mask drawn on the original IHC image to exclude areas of tissue folding or defects was after rescaling overlaid on the AR images, rescaled brown IHC image and rescaled blue IHC image before data extraction. Furthermore the rescaled brown IHC and blue IHC images were dichotomized to be either positive (1) or negative (0) for the respective stains before data extraction. The corresponding values of Ki-67 immunostaining relative to blue nuclear staining (number of positive brown pixels/number of positive blue pixels) and of tracer uptake (counts) were evaluated for areas of 10 x 10 pixels (420 μm x 420 μm) at a time by taking the average. Pixels lining the edges of the mask delineating the tissue of interest were excluded during this procedure and an equal cell distribution within the examined tissue of interest was assumed. Hereby correlations between Ki-67 IHC as a marker of proliferation and tracer uptake of ^64^Cu-ATSM (hypoxia) or ^18^F-FDG (glycolysis) could be established.

### Gamma counting and calculation of a standardized uptake value of ^64^Cu-ATSM and ^18^F-FDG for each tumour biopsy

The 30–45 tumour pieces (approximately 5 x 5 x 5 mm) from each tumour, to be evaluated by gamma counting and qPCR, were individually placed in pre-weighed Eppendorf tubes containing RNA*later* (Ambion Inc. Austin, TX, USA). Tubes were weighed and using a WIZARD^2^® Automatic gamma counter (model 2480, PerkinElmer, Waltham, MA, USA) gamma counting of each tumour piece was performed twice approximately 19 hours apart. Thereby the first gamma count would be a mixed count of ^64^Cu-ATSM and ^18^F-FDG, while the second count represented solely ^64^Cu-ATSM. Empty Eppendorf tubes were counted to correct for the background count rate. The counting efficiency of the equipment was 9.43 x 10^−2^ for ^64^Cu-ATSM and 0.54 for ^18^F-FDG.

Based on the gamma counting data, the matrix functions MMINVERSE and MMULT (Microsoft^®^ Excel^®^ for mac 2011, version 14.3.8, Microsoft Corporation, Remond, WA, USA) were applied to solve the following two equations with two unknowns with regard to $A_{0_{64_{\text{Cu-ATSM}}}}$ and $A_{0_{18_{\text{F-FDG}}}}$ for each tumour piece to find the activity of ^64^Cu-ATSM and ^18^F-FDG.

$$R_{s}1\;=\;R_{T}1-R_{B}1\;=\;\varepsilon_{18_{\text{F-FDG}}}\cdot A_{0_{18_{\text{F-FDG}}}}+\varepsilon_{64_{\text{Cu-ATSM}}}\cdot A_{0_{64_{\text{Cu-ATSM}}}}$$

$$R_{s}2=\varepsilon_{18_{\text{F-FDG}}}\cdot A_{0_{18_{\text{F-FDG}}}}\cdot e^{{-\lambda }_{18_{\text{F-FDG}}}\cdot t_{2}}+\varepsilon_{64_{\text{Cu-ATSM}}}\cdot A_{0_{64_{\text{Cu-ATSM}}}}\cdot e^{{-\lambda }_{64_{\text{Cu-ATSM}}}\cdot t_{2}}$$

*R*_*S*_1, *R*_*T*_1 and *R*_*B*_1is the net count rate, total count rate and background count rate for the first gamma count, respectively, while *R*_*S*_2 is the net count rate for the second gamma count. $\varepsilon_{64_{\text{Cu-ATSM}}}$ and $\varepsilon_{18_{\text{F-FDG}}}$ are the counting efficiencies for ^64^Cu-ATSM and ^18^F-FDG respectively, while $\lambda_{64_{\text{Cu-ATSM}}}$ and $\lambda_{18_{\text{F-FDG}}}$ are their decay constants. *t*_2_ represents the time between the beginning of the two gamma counts for a given tumour piece. Finally $A_{0_{64_{\text{Cu-ATSM}}}}$ and $A_{0_{18_{\text{F-FDG}}}}$ are the activities of ^64^Cu-ATSM and ^18^F-FDG at the time of the first gamma count for a given tumour piece. These activities were converted to a standardized uptake value (SUV) by taking the weight of the tumour piece, the canine patient’s body weight (BW) and the injected dose into consideration [47, 48]:

$$SUV\;=\frac{\text{Activity in tumour piece}\;(kBq/ml)}{\text{Injected tracer activity}\;(MBq)/\text{BW}\;(kg)}$$

### Quantitative real-time polymerase chain reaction

The gamma counted tumour pieces were stored in RNA*later* for approximately 24 hours at 4°C before removing the supernatant and storing the tissue at -80°C until use.

#### Total RNA extraction and reverse transcription

RNAzol^®^RT (Molecular Research Center Inc., Cincinnati, OH, USA), including the 4-bromoanisole (BAN) step to further eliminate genomic DNA contamination, was used according to the manufacturer’s protocol to extract total RNA from each tumour piece (13.4–510.9 mg). The tissue was lysed and homogenized in Precellys^®^-24 (Bertin Technologies, Montigny, France).

The total RNA concentration was determined by NanoDrop 1000 (Thermo Fisher Scientific, Waltham, MA, USA) to be between 262.4 and 4368.3 ng RNA/μL. To further minimize genomic DNA contamination, all RNA samples were treated with rDNAse (Macherey-Nagel, Düren, Germany) according to the manufactures’ protocol, except for the incubations which were performed at 75°C for 15 minutes and at 4°C for 15 minutes. The quality of the isolated RNA was measured as the RNA Integrity Number (RIN) on a 2100 Bioanalyzer (Agilent Technologies, Santa Clara, CA, USA). RIN values were between 5.4 and 9, verifying good RNA quality [49].

Total RNA (0.3 μg) was reverse transcribed (RT) using AffinityScript^TM^ QPCR cDNA synthesis kit (cat.no. 600559, Stratagene, Santa Clara, CA, USA): Seven μL RNA (0.3 μg) + 2.45 μL oligo (dT) primer (0.1 μg/μL) + 0.55 μL random primer (0.1 μg/μL) + 10 μL First Strand Master Mix (2x) + 1 μL StrataScript RT-Rnaseblock; ending up with a final volume of 21 μL cDNA. RT was performed on a MasterCycler Gradient (Eppendorf AG, Hamburg, Germany): incubation at 25°C for 5 minutes (primer annealing), 42°C for 15 minutes (cDNA synthesis) and 95°C for 5 minutes (termination of cDNA synthesis). Immediately after the cDNA was cooled down, frozen and stored at -20°C.

#### Primers and TaqMan dual-labelled probes

Canine *β-*Glucuronidase (c_GUSB, NM_001003191) and proteasome subunit, beta type, 6 (c_PSMB6, XM_844148) were selected as reference genes, based on our previous study of suitable reference genes in similar tissues [50]. In brief this study demonstrated how to identify suitable reference genes for normalization of genes of interest (GOIs) in canine soft tissues sarcomas using qPCR and report these according to the MIQE guidelines [51]. Primer pairs for 17 potential reference genes were designed and investigated in archival tumour biopsies from 6 canine soft tissue sarcomas by quantifying the gene expression in duplicate samples run in SYBRGreen I. geNorm [52] implemented in the qBase^PLUS^ software [53] (Biogazelle NV, Zwijnaarde, Belgium) was used to identify the optimal number of reference genes and determine these.

In the present study GUSB and PSMB6 were designed in a TaqMan duplex assay while GOIs were designed as multiplex (duplex or triplex) or simplex TaqMan assays. The following GOIs were investigated: c_Ki-67 (XM_005637893) indicating proliferation; c_CAIX (NM_001145174) and c_HIF-1α (XM_003639201) as measures of hypoxia; c_TF (NM_001024640) and c_VEGFA (NM_0010003175) representing angiogenesis and c_HK2 (XM_003639587), c_GLUT1 (NM_001159326) and c_GLUT3 (NM_0010003308) as indicators of glycolysis.

Beacon Designer (version 8.02–8.12, Premier BioSoft, Palo Alto, CA, USA) was used to design primers and probes. Before designing, all genes were tested for cross homology against the canine genome and checked for secondary structures. Since HIF-1α exists in 2 transcript variants and VEGFA in 3, the amplicons for these two targets were placed in a way including all transcript variants. The optimized conditions for the primers and probes, after testing and securing one melting product and an efficiency of 100 ± 10%, are listed in Table 2. Primers and probes were purchased from Sigma-Aldrich (St. Louis, MO, USA). Final qPCR designs are listed in Table 3.

**Table 2:** Optimized primer- and TaqMan probe concentrations for the genes investigated.

Gene	Forward primer Final concentration (nM)	Reverse primer Final concentration (nM)	TaqMan probe Final concentration (nM)
**Reference genes**			
c_GUSB (*GUSB*)	300	600	300
c_PSMB6 (*PSMB6*)	300	300	250
**Genes of interest (GOIs)**			
c_Ki-67 (*MKI67*)	300	600	300
c_CAIX (*CA9*)	600	600	200
c_HIF-1α (*HIF1A)*	600	300	250
c_TF (*TF*)	300	600	250
c_VEGFA (*VEGFA*)	600	300	300
c_GLUT1 (*SLC2A1*)	300	600	150
c_HK2 (*LOC100856448*)	300	300	250
c_GLUT3 (*SLC2A3*)	600	600	300

**Table 3:** Final qPCR designs for the reference genes and the genes of interest (GOIs).

Gene	Forward primer (5'-3')	Reverse primer (5'-3')	5'-Flourophore	TaqMan probe, 5'-3'	3'-Quencher	Amplicon length (bp)
c_GUSB	GTCCTCCTGCCGTATTAC	CGTAGTTGAAGAAGTCAAAGTA	HEX	CTTGCCATCAACAACACGCTCAC	BHQ1	111
c_PSMB6	TGCAGAATCAGGGGTAGA	TCGCTTACTGTAGCCTAG	FAM	AGACCAGATTCCCAAATTCACCATCG	BHQ1	146
c_Ki-67	CAGCAAATCTCCTCATCA	CTTCGATCAATGGAAGTTC	FAM	CAAGTTGCCGCTCCTCTTCC	BHQ1	90
c_CAIX	CTTGGAACTTGGAGAATATG	GGAAGTGGTATAAAGGGTA	FAM	AGAGAAGCCAACCAGAAGAATCT	BHQ1	94
c_HIF-1α	CCACAACATCACCATACA	TTCCGTCTGTTCTATTACTC	FAM	AAGTCGGACAGCCTCACCAA	BHQ1	83
c_TF	TCCTCGTCATTGGAATTG	CTTCCTGCACTTGTACAG	HEX	ATCATCTTCACCATCATCCTGTCTGT	BHQ1	80
c_VEGFA	GGTCTTTGTGTTTAAGATTCA	GAGTGTTAGCAAAATTAAATATCTG	Cy5	CTCTCTCCCTGATCGGTGACAG	BHQ2	98
c_GLUT1	CACACTAATCGAACTATGAACTA	GTTGGGCAGGAAGAGATG	FAM	CGAACCCTAATGGAGCCTGACCCT	BHQ1	138
c_HK2	CCCCACTTTAAATTATAAGATGTC	AAGGCTTGGGGATTGAAC	HEX	ATCCACAGACAGCACACCCAGT	BHQ1	133
c_GLUT3	GCTACTTGATTCCTTTCTC	GTTCCTCCTGAAATGAAG	FAM	ACACTCCATGAGCACTCAGAAGAA	BHQ1	158

Primer and probe sequences, the flourophores and quenchers used for the TaqMan probes and the amplicon length (in base pairs (bp)) of the product for each gene are listed. Reference genes GUSB (NM_001003191) and PSMB6 (XM_844148) were designed in a duplex assay. GOIs Ki-67 (XM_005637893), CAIX (NM_001145174) and GLUT3 (NM_0010003308) were designed in simplex assays; GOIs HIF-1α (XM_003639201), TF (NM_001024640) and VEGFA (NM_0010003175) in a triplex assay and GOIs HK2 (XM_003639587) and GLUT1 (NM_001159326) in a duplex assay.

#### qPCR

Gene expression was quantified on the Mx3005P real-time PCR systems from Stratagene (Santa Clara, CA, USA) using Brilliant III Ultra-Fast QPCR Master Mix (Stratagene, cat. no. 600880) for the simplex assays and Brilliant Multiplex QPCR Master Mix (Stratagene, cat. no. 600553) for the multiplex assays. The thermal profile for Brilliant III Ultra-Fast reagent was: 3 minutes of denaturation at 95°C, followed by 40 cycles with denaturation at 95°C for 20 seconds and annealing/elongation at 60°C for 20 seconds. For the Brilliant Multiplex reagent, the thermal profile was: denaturation at 95°C for 10 minutes, followed by 40 cycles with denaturation at 95°C for 15 seconds and annealing/elongation at 60°C for 1 minute.

Samples were run in duplicates using 1 μL cDNA in a total volume of 20 and 25 μL, respectively. A duplicate NO RT (no reverse transcription), indicating contamination with genomic DNA in the sample, was included for each sample using a dilution of the respective sample mRNA. Furthermore a NTC (no template control) and a dilution curve, used for efficiency calculations, were included alongside three inter-run calibration samples in each run.

#### qPCR data analysis

qBase^PLUS^ software based on the comparative 2^-ΔΔCq^ method [54] for relative quantification was used to analyse the qPCR data for each tumour individually. Samples with the following criteria were excluded from the analysis: a) a difference in Cq value between the sample and the corresponding NO RT of less than 5 Cq, b) a replicate variability difference in Cq value of more than 0.5 Cq. Normalization to two reference genes was included based on the findings in our previous study [50]. The software also calculated the stability of the two selected reference genes. We only accepted a reference target stability value M below 1.44 and a CV-value below 0.58 due to the expected heterogeneous composition of the tumour biopsies including both stromal, endothelial and tumour cells. Hereby we excluded patient 1 from further qPCR analysis. Furthermore the qBase^PLUS^ software accounted for the three inter-run calibrators for correction between PCR runs within the same gene. The data were reported as calibrated normalized relative quantities (CNRQs).

### Statistical analysis

Tumour piece uptake of ^64^Cu-ATSM and ^18^F-FDG calculated as SUV from gamma counts were correlated to each other and the gene expression of GOIs using Spearmann’s rank correlation in Graph Pad Prism (version 6.0e, GraphPad Software, Inc., La Jolla, CA, USA). MATLAB (version R2014a, The Mathworks, Inc., Natick, MA, USA) was used for correlating ^64^Cu-ATSM and ^18^F-FDG AR images to each other. Processing of data from IHC images and AR images was done using Excel^®^ (Microsoft^®^ Excel^®^ for mac 2011, version 14.3.8, Microsoft Corporation, Redmond, WA, USA) and Graph Pad Prism (version 6.0e, GraphPad Software, Inc., La Jolla, CA, USA) to calculate Spermann’s rank correlations between Ki-67 immunostaining and ^64^Cu-ATSM and ^18^F-FDG in tumour tissue sections. For all statistical analyses a p-value < 0.05 was considered significant.

## Results

### Micro regional uptake, distribution and correlation of ^64^Cu-ATSM and ^18^F-FDG

**Fig 1:**
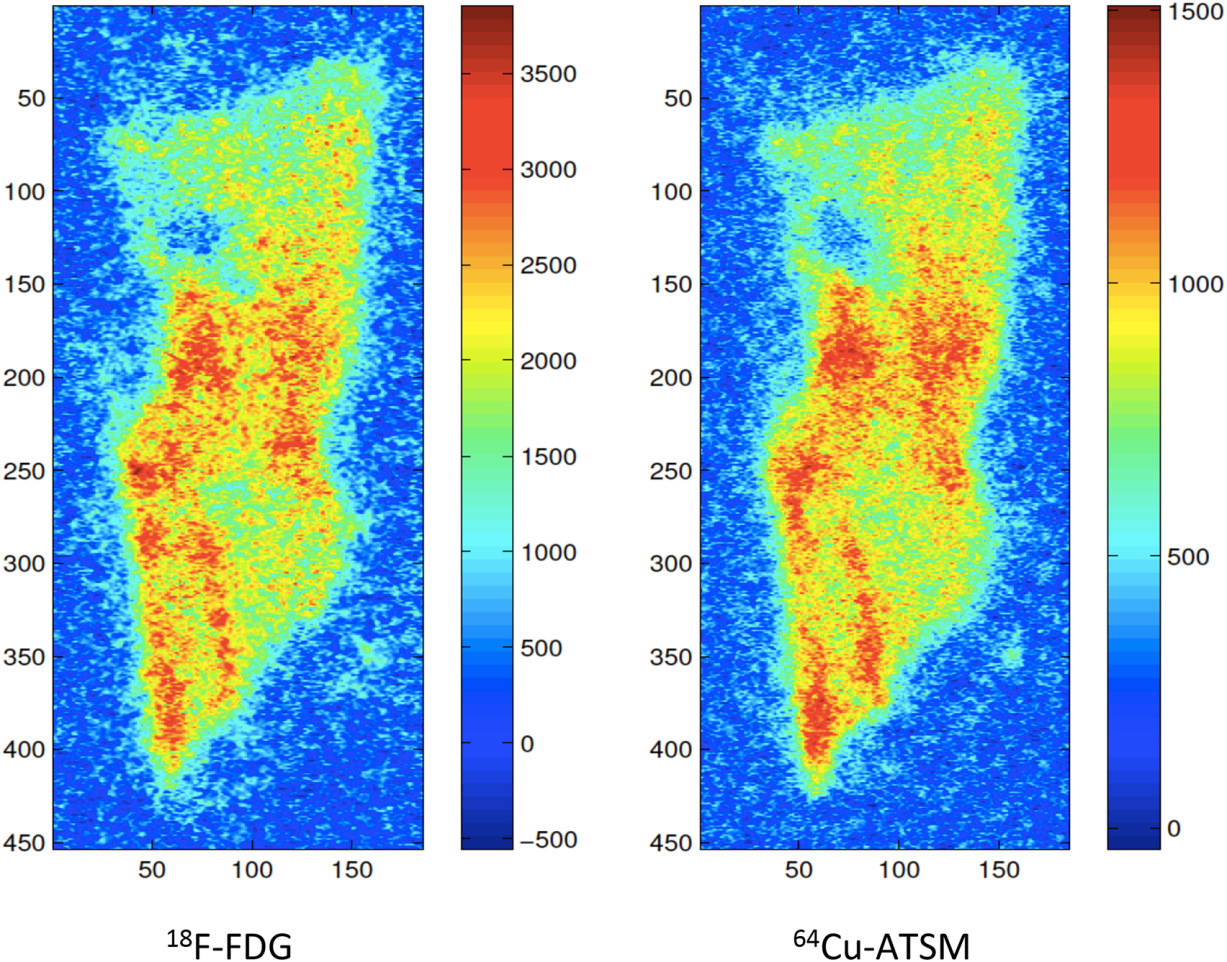
Micro regional heterogeneity in distribution of ^64^Cu-ATSM (hypoxia) and ^18^F-FDG (glycolysis). Example of calculated ^18^F-FDG image (left) and calculated ^64^Cu-ATSM image (right) from autoradiography of a tumour tissue section from tumour 4. Intensity levels in each image are individually optimized.

**Fig 2:**
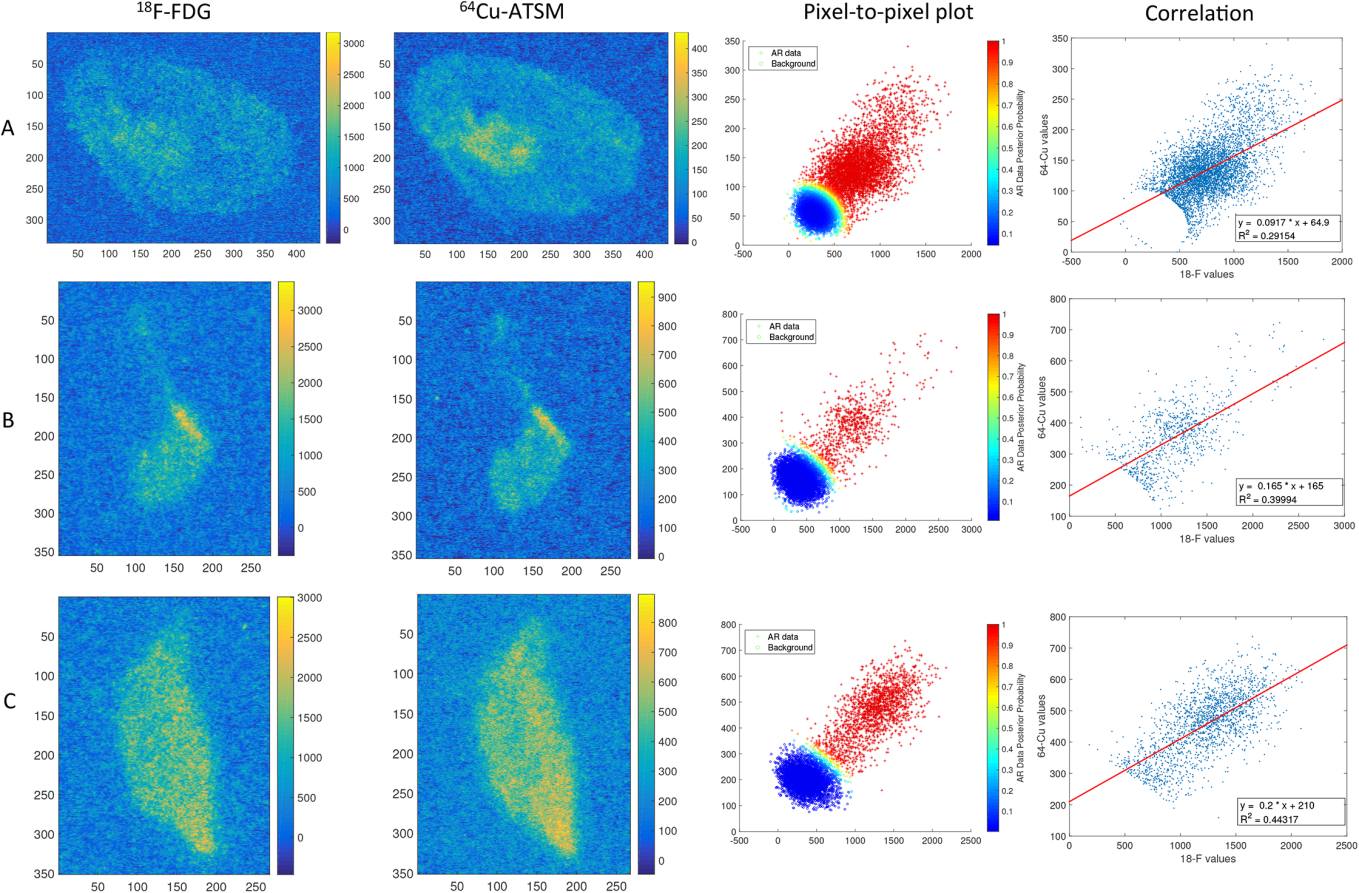
Correlation between ^64^Cu-ATSM and ^18^F-FDG distribution in autoradiography sections. Examples of calculated ^18^F-FDG images (first column) and calculated ^64^Cu-ATSM images (second column) from the autoradiographies from tumour 2 (**A**), tumour 3 (**B**) and tumour 4 (**C**). The third column shows the pixel-to-pixel plot, separating background (blue marks) and autoradiography (AR) image data (red marks) using cluster analysis. Images were downscaled a factor of 4 for this analysis. The correlation of the non-background AR image is shown in the fourth column.

**Fig 3:**
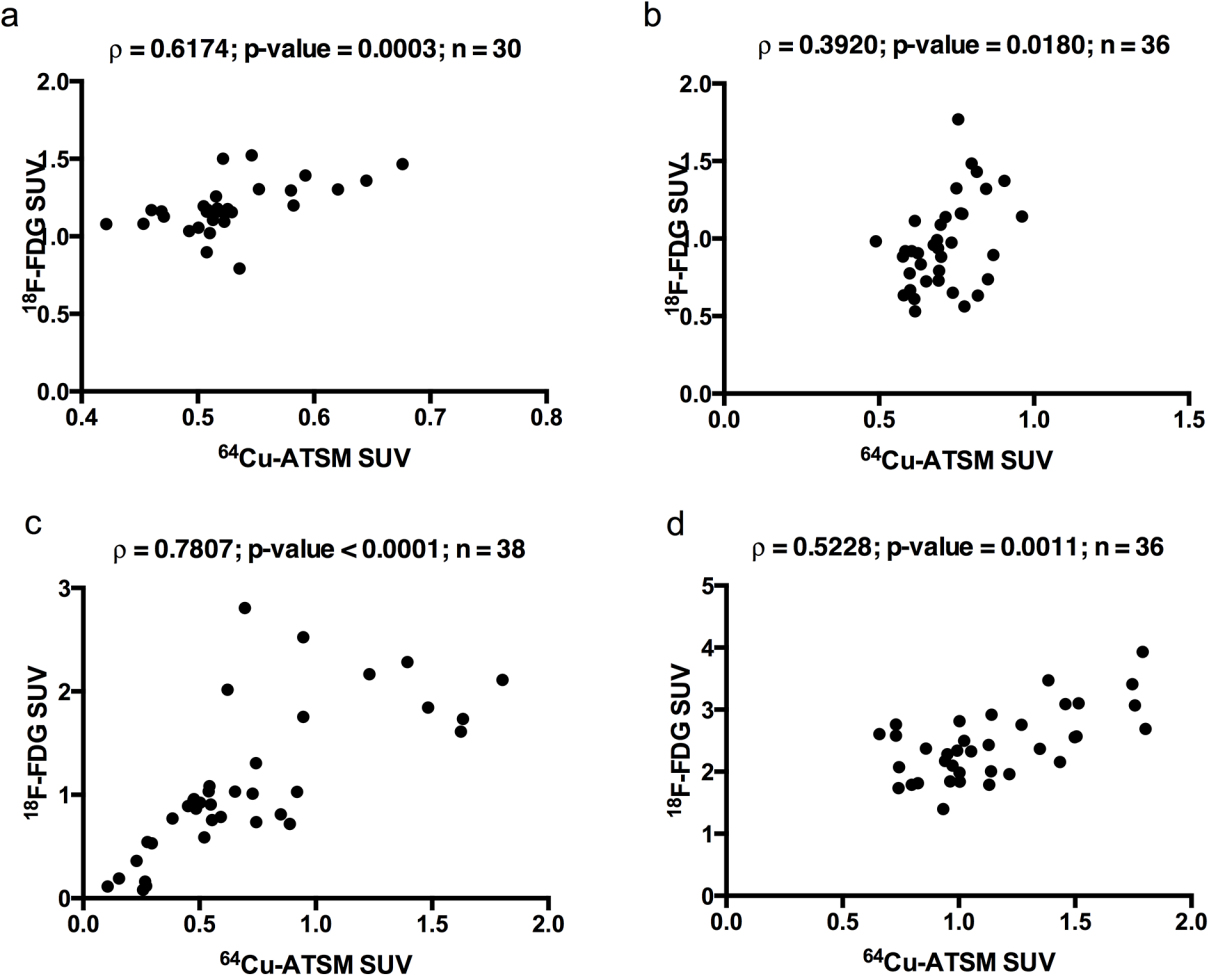
Correlation between ^64^Cu-ATSM and ^18^F-FDG uptake in gamma counted tumour pieces. Spearman’s Rank correlations (ρ) and p-values for comparison of intra-tumoural spatial distribution of ^64^Cu-ATSM and ^18^ F-FDG calculated as standardized uptake value (SUV) from gamma counts of tumour pieces. **a**, **b**, **c** and **d** show data for tumour pieces from canine cancer patient 1, 2, 3 and 4, respectively. n is the number of tumour pieces included in the final analysis. Significant correlations are written in bold.

As exemplified in Fig 1 varying degrees of micro regional tumour heterogeneity in hypoxia, as per ^64^Cu-ATSM uptake, and glycolysis, as per ^18^F-FDG uptake, within tumour tissue sections from each tumour was observed. Visual examination of AR images of ^64^Cu-ATSM and ^18^F-FDG likewise revealed different degrees of micro regional heterogeneity in the uptake of the two tracers between distinct tumour tissue sections from each individual tumour as well as between the four examined sarcomas. However the spatial distribution of ^64^Cu-ATSM and ^18^F-FDG was rather similar within each tumour tissue section, which was also reflected in the positive correlations found in all AR images between the spatial distribution of ^64^Cu-ATSM and ^18^F-FDG based on pixel-to-pixel analysis (Fig 2). Similarly, when correlating the SUVs of ^64^Cu-ATSM and ^18^F-FDG for the multiple tumour pieces within each individual tumour, a moderate positive significant correlation (*ρ* between 0.3920 and 0.7807; p between 0.0180 and <0.0001) between the two tracers was seen in all four sarcomas (Fig 3).

Overall SUVs for each of the 30–45 pieces from the individual tumours calculated from the gamma counts were generally lower for ^64^Cu-ATSM than for ^18^F-FDG for all four tumours (Table 4). Furthermore ^64^Cu-ATSM SUVs showed less variation between the individual tumours compared to ^18^F-FDG SUVs (Table 4).

**Table 4:** Maximum and minimum standardized uptake values (SUV) for ^64^Cu-ATSM and ^18^F-FDG in each tumour calculated from well counts on all biopsies.

	^18^F-FDG SUV		^64^Cu-ATSM SUV	
Tumour no.	Minimum	Maximum	Minimum	Maximum
1	0.79	1.52	0.42	0.68
2	0.53	1.77	0.49	0.96
3	0.08	2.81	0.10	1.80
4	1.40	3.93	0.66	1.80

### Ki-67 Immunohistochemistry: relation to ^64^Cu-ATSM and ^18^F-FDG autoradiography

**Fig 4:**
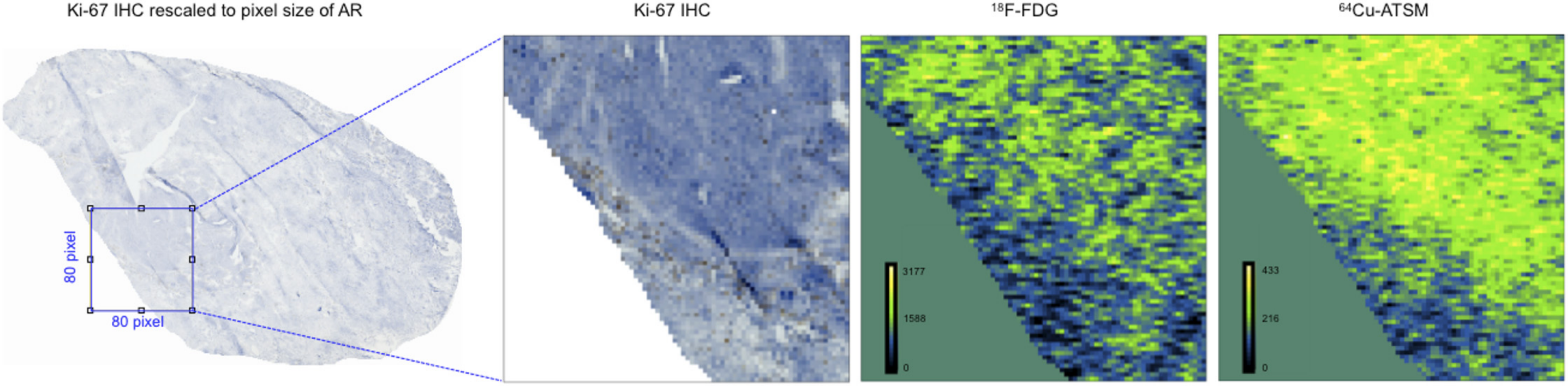
Ki-67 IHC versus ^64^Cu-ATSM and ^18^F-FDG autoradiography. Visual example of the comparison between a Ki-67 IHC image and ^64^Cu-ATSM and ^18^F-FDG autoradiography images for tumour 2. First column: Ki-67 IHC image rescaled to the same pixel size (42 μm) as the autoradiographies (AR). A selection is chosen for illustration of correlations between Ki-67 IHC and ^18^F-FDG and ^64^Cu-ATSM AR respectively (columns 2–4).

The proliferative state of the tumours, investigated through Ki-67 IHC, revealed an overall scarce Ki-67 staining with a rather homogenous distribution without any obvious hotspots within the tumour sections. When compared with the uptake of ^64^Cu-ATSM and ^18^F-FDG of the corresponding AR images only for tumour 3 a significant but negative correlation was found between Ki-67 IHC and ^64^Cu-ATSM (*ρ* = -0.5901; p = 0.0006). The remainder of the correlations were non-significant (p between 0.2123 and 0.3663) or irrelevant (*ρ* between -0.1433 and -0.1949). A visual example of the comparison between a Ki-67 IHC image and ^18^F-FDG and ^64^Cu-ATSM AR images is given in Fig 4.

### Genes of interest: Relation to ^64^Cu-ATSM and ^18^F-FDG uptakes

**Fig 5:**
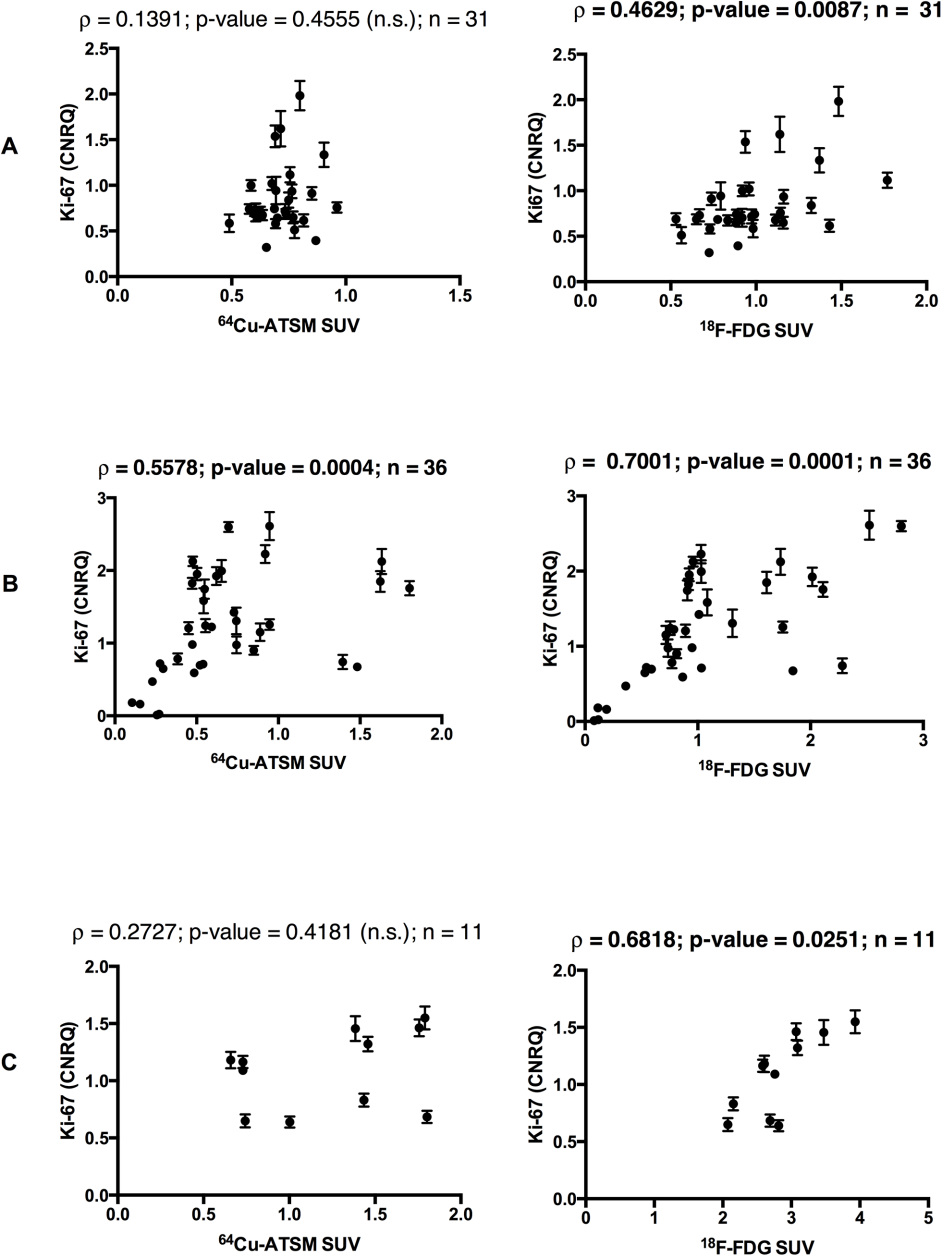
Correlation between gene expression of Ki-67 and ^64^Cu-ATSM and ^18^F-FDG uptake. Spearman’s Rank correlations (ρ) and p-values for comparison of gene expression for Ki-67 and tumour uptake of ^18^F-FDG (right column) and ^64^Cu-ATSM (left column) calculated as standardized uptake value (SUV) from gamma counts of tumour pieces. Row **A**, **B** and **C** show data for tumour pieces from canine cancer patient 2, 3 and 4 respectively. n is the number of tumour pieces included in the final analysis. *n*.*s*. not significant. Significant correlations are written in bold.

In contrast to the Ki-67 IHC results, the pairwise measurements of relative gene expression and radioactivity for the multiple tumour biopsies within each individual tumour revealed significant positive correlations between Ki-67 gene expression and the uptake of ^18^F-FDG in all three tumours (Fig 5, right column), while this was only the case for one tumour (no. 3) when correlating to the uptake of ^64^Cu-ATSM (Fig 5, left column).

**Fig 6:**
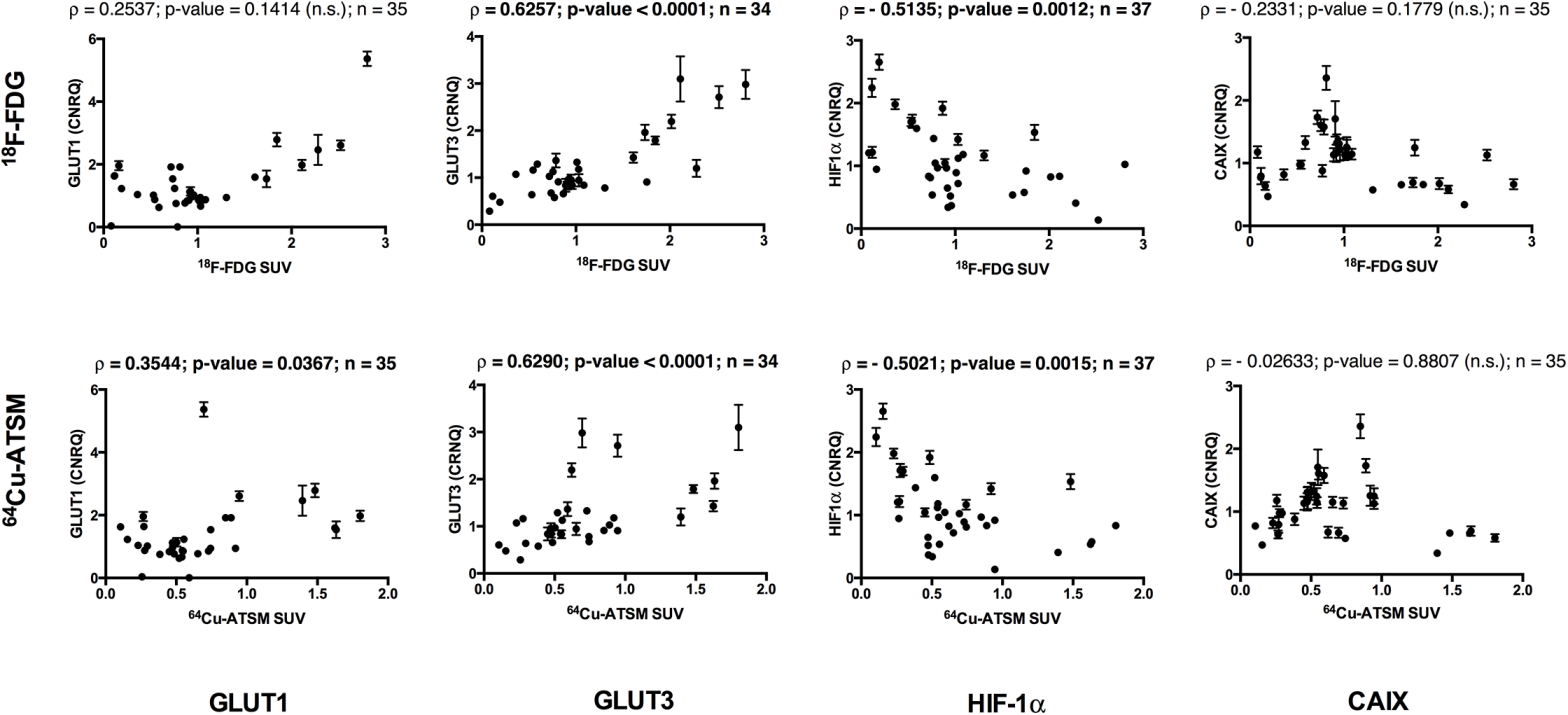
Correlations between hypoxic and glycolytic gene expressions and ^64^Cu-ATSM and ^18^F-FDG uptake, tumour 3. Spearman’s Rank correlations (ρ) and p-values for comparison of gene expressions for GLUT1, GLUT3, HIF-1α and CAIX respectively and tumour uptake of ^18^ F-FDG (upper row) and ^64^Cu-ATSM (lower row) calculated as standardized uptake value (SUV) from gamma counts. n is the number of tumour pieces included in the final analysis. *n*.*s*. not significant. Significant correlations are written in bold.

Results from this tumour, regarding correlations of tracer uptake to hypoxic and glycolytic gene expression markers, also demonstrated significance for both ^64^Cu-ATSM and ^18^F-FDG (Fig 6). The gene expression of HIF-1α exhibited a significant negative correlation to both ^18^F-FDG (*ρ* = -0.5135; p = 0.0012) and ^64^Cu-ATSM (*ρ* = -0.5021; p = 0.0015), while the correlations between the tracers and CAIX gene expression were non-significant. With regard to the expression of GLUT1 and GLUT3 both ^64^Cu-ATSM and ^18^F-FDG showed positive correlations, though not significant for GLUT1 versus ^18^F-FDG (*ρ* = 0.2537; p = 0.1414) (Fig 6). For the angiogenic gene expression markers the correlations to tracer uptake were non-significant for tumour 3, except for a positive significant correlation between ^18^F-FDG and TF (*ρ* = 0.4885; p = 0.0046). Similarly, non-significant correlations were seen in the two other tumours (no. 2 and 4) for some of the investigated GOIs and no general trend was found within the significant correlations (S1 Fig).

**Fig 7:**
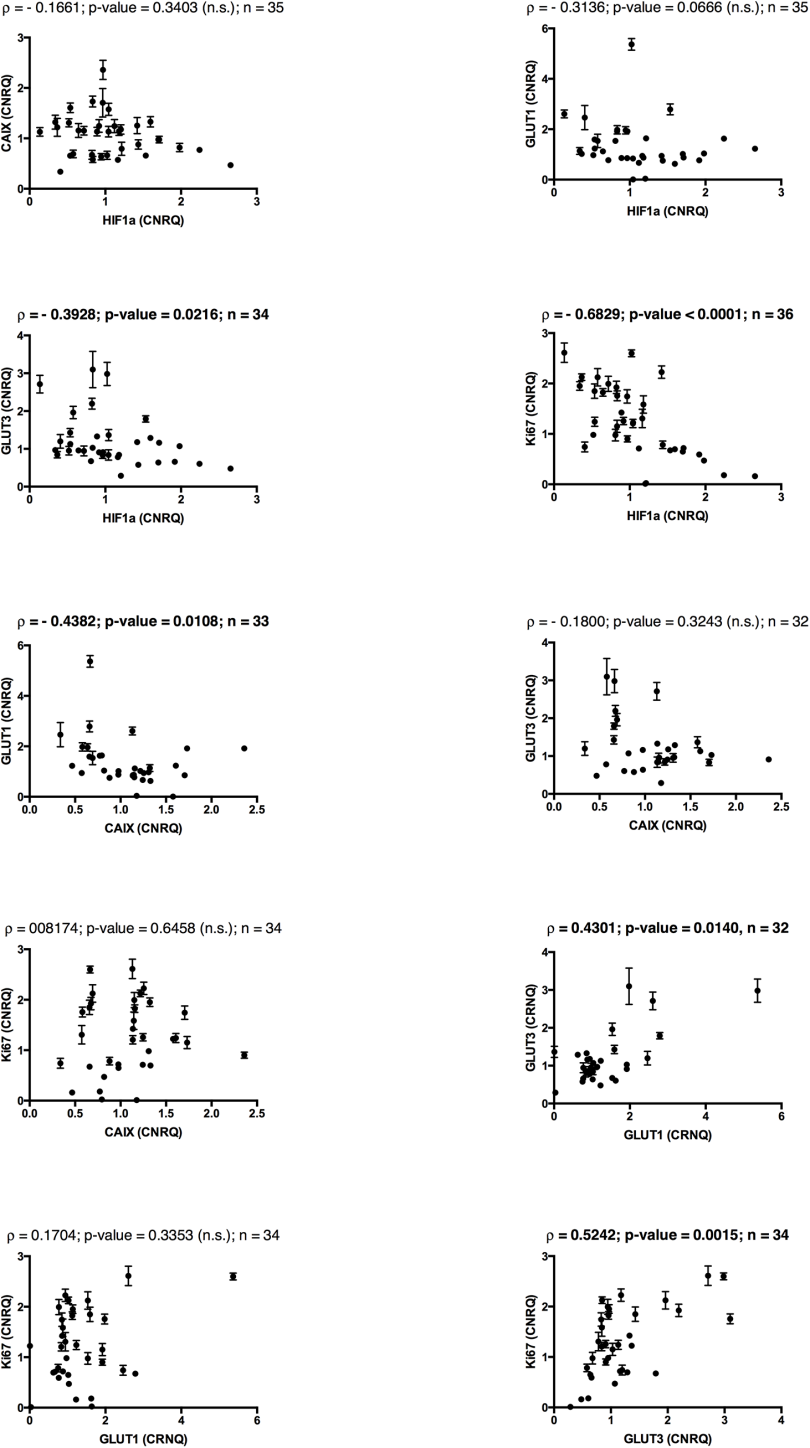
Correlations between the gene expressions of different genes of interest, tumour 3. Spearman’s Rank correlations (ρ) and p-values for all possible gen-gen correlations between GLUT1, GLUT3, HIF-1α, CAIX and Ki-67. n is the number of tumour pieces included in the final analysis. *n*.*s*. not significant. Significant correlations are written in bold.

To further elucidate the significant correlations between the tracers and gene expressions found in tumour 3, we correlated the gene expression of different GOIs to each other (Fig 7).

## Discussion

This unique dual tracer study exploited the different half-lives of ^64^Cu and ^18^F to simultaneously investigate the micro regional heterogeneity of tumour microenvironment hypoxia and glycolysis in canine soft tissue sarcomas using the PET tracers ^64^Cu-ATSM and ^18^F-FDG and their relation to endogenous markers of hypoxia, glycolysis, proliferation and angiogenesis. Importantly, it was for the first time in spontaneous canine soft tissue sarcomas simultaneously demonstrated, that the uptake and spatial distribution of each of the tracers ^64^Cu-ATSM and ^18^F-FDG showed micro regional distribution differences, while the two tracers showed varying degrees of overlapping micro regional distributions of hypoxia and glycolysis manifested in significant moderate correlations between ^64^Cu-ATSM and ^18^F-FDG examined both through gamma counting and autoradiographies. This demonstrates both some overlap but also uniqueness of each of the PET tracers. The expression of the endogenous markers of hypoxia and glycolysis showed similar significant correlations to both ^64^Cu-ATSM and ^18^F-FDG in one tumour, while no particular trend was observed in the other tumours or for the endogenous markers of angiogenesis potentially indicating differing molecular expression patterns in the examined soft tissue sarcomas. With regard to tumour cell proliferation, gene expression of Ki-67 and tracer uptake for ^18^F-FDG correlated closer than Ki-67 and ^64^Cu-ATSM, indicating that ^18^F-FDG may be a better marker of proliferation.

### Micro regional uptake, distribution and correlation of ^64^Cu-ATSM and ^18^F-FDG

As stated above the present study contributes with novel information about tumour heterogeneity of hypoxia, as per ^64^Cu-ATSM uptake, and glycolysis, as per ^18^F-FDG uptake, at the micro regional level. It is innovative in simultaneously investigating two PET tracers in a spontaneous syngeneic translational animal cancer model and performing pairwise comparisons of tracer uptake with endogenous markers in small tumour pieces. As hypothesized a moderate significant correlation was found between the uptake and spatial distribution of ^64^Cu-ATSM and ^18^F-FDG.

Other studies have also investigated the spatial overlap of ^64^Cu-ATSM and ^18^F-FDG using either AR or PET image comparisons. Comparable to the results of the present study, a previous canine study including both sarcomas and carcinomas reported moderate to strong spatial correlations between ^64^Cu-ATSM (3 and 24 hours post injection) and ^18^F-FDG based on voxel comparisons of PET images, though without specifying the correlations according to histopathology or uptake in multiple tumour pieces as in the present study [39]. In a similar cohort comparing ^64^Cu-ATSM and ^18^F-FDG PET scan uptakes with immunohistochemical staining for the exogenous hypoxia marker pimonidazole, it was found that tumours with high levels of pimonidazole staining showed high uptake of ^64^Cu-ATSM (3 hours post injection) and ^18^F-FDG [39]. Furthermore comparison of ^64^Cu-ATSM AR (25 hours post injection) and pimonidazole IHC revealed similar regional distributions in the most heterogeneous tumour regions. ^18^F-FDG AR however was not available for comparison with pimonidazole and the study concluded that ^18^F-FDG might not be a surrogate marker of hypoxia [55]. In partial contrast the findings of moderate spatial micro regional overlap between ^64^Cu-ATSM and ^18^F-FDG in the present study, a recent study in human patients with head and neck cancer showed no significant difference in the biological tumour volume delineated for radiotherapy planning based on ^64^Cu-ATSM PET/CT or ^18^F-FDG PET. However the correlation analysis was not described in detail [40]. A newer PET study of canine sinonasal cancers did not find as strong spatial correlations between ^61^Cu-ATSM and ^18^F-FDG for sarcomas as it did for carcinomas [56], suggesting that tumour type differences may exist. The present study cannot evaluate this, as only canine soft tissue sarcomas were examined. However earlier rodent model studies also revealed differences in ^64^Cu-ATSM uptake between various cancers [57].

In contrast to the results of the present study, poor correlations between the micro regional distribution of ^64^Cu-ATSM and ^18^F-FDG evaluated by AR have been seen in preclinical studies in both mouse [41, 42], rat [43, 44] and rabbit tumour models [45]. A very recent study using ^64^Cu-ATSM and ^18^F-FDG PET/CT scans in canine spontaneous carcinomas and sarcomas for dose painting also revealed varying degrees of correlation between dose plans based on the two tracers [58]. Furthermore it was found that ^18^F-FDG based dose painting plans only covered approximately 50% of the hypoxic regions adequately, leading to the conclusion that ^64^Cu-ATSM and ^18^F-FDG provide different biological information [58]. In agreement with this, the present study showed only a moderate overlap between ^64^Cu-ATSM and ^18^F-FDG in canine soft tissue sarcomas, but with the novelty of being evaluated simultaneously and at the micro regional level. In addition, the present study revealed varying degrees of heterogeneous distribution of each individual tracer within and between tumour tissue sections from each tumour, indicating the existence of tumour heterogeneity in hypoxia and glycolysis at the micro regional level. In sum, in some circumstances ^64^Cu-ATSM and ^18^F-FDG may be closely related but this is not always the case. Therefore, both tracers have their value when phenotyping tumours and ^64^Cu-ATSM is justified as an adjunct to ^18^F-FDG imaging.

### Gene expression of endogenous markers of hypoxia, glycolysis and angiogenesis in relation to ^64^Cu-ATSM and ^18^F-FDG uptakes calculated from gamma counts

The multiple pairwise measurements of radioactivity attributable to ^64^Cu-ATSM and ^18^F-FDG respectively and the gene expressions of endogenous markers in small tumour pieces helped elucidate the true connections between tracer uptake and molecular events on a micro regional level and gave insights into micro regional heterogeneity. Significant correlations between the gene expression of endogenous markers of hypoxia and glycolysis and the PET tracer uptakes were seen with similar correlations for both ^64^Cu-ATSM and ^18^F-FDG in one tumour (no. 3), while no particular trend was seen for the endogenous markers of angiogenesis or in the other tumours, indicating potential differences in molecular expressions in the investigated tumours.

The correlations between tracer uptake and gene expression in tumour 3 revealed a positive correlation between ^18^F-FDG and the mRNA encoding the glucose transporters GLUT1 and GLUT3 as expected, though only significant for GLUT3. GLUT1 and GLUT3 are the primary transporters facilitating uptake of glucose and thus also the glucose analogue ^18^F-FDG into the tumour cells [59–61]. That ^18^F-FDG uptake only correlated significantly with GLUT3 may be explained by the higher affinity for glucose and the greater transport capacity of this glucose transporter compared to GLUT1 [62]. However it may also indicate that this transporter has the main responsibility for glucose transport into cells in canine soft tissue sarcomas or be due to post transcriptional or translational changes. The significant positive correlation seen between ^64^Cu-ATSM and GLUT1 and especially GLUT3 reflects the close link between hypoxia and increased glycolysis in tumours. Both GLUT1 and GLUT3 are known to be up-regulated by HIF-1α during hypoxia [63, 64]. Interestingly enough we did not find a positive but rather a negative correlation when comparing the gene expression of GLUT3 and, though not significant, GLUT1 with HIF-1α gene expression in tumour 3. This discrepancy may be explained by post transcriptional or translational modifications in HIF-1α, remembering that the HIF-1α protein is the active transcription factor for hypoxia inducible genes [65, 66]. Another explanation for the negative correlations between HIF-1α and its target genes could be the existence of acute or recent hypoxia within the cells leading to up-regulation of HIF-1α while the transcription of the hypoxia responsive genes has not yet been significantly up regulated.

We also found negative correlations between both ^64^Cu-ATSM and ^18^F-FDG with regard to the gene expression of CAIX (though non-significant) and HIF-1α in tumour 3. For ^18^F-FDG this seems reasonable, since this tracer is primarily a marker of tumour glycolysis. However since the correlations were equal for ^64^Cu-ATSM and ^18^F-FDG a similar explanation for the negative correlation for the two PET tracers may also be possible. One reason could be the above-described delay in transcription of hypoxia responsive genes in areas of acute hypoxia. Another explanation could be that HIF-1α is regulated by other factors than tumour hypoxia, such as oncogenic signalling or growth factors [67–69]. Probably this also explains why a lack of correlation between hypoxia measurements and expression of HIF-1α and its target genes have been seen not only in some of the tumours in the present study, but also in other studies [70, 71]. Similarly, negative or non-significant correlations between CAIX and hypoxia measurements have also been found in other studies [72].

Different explanations can be put forward to justify that tumour 3 showed significant correlations between tracer uptake and gene expression with a noticeable trend for both tracers, while more non-significant correlations and no specific trends were seen for the correlations in the other sarcomas. The most plausible reason is the wider range of SUVs for both ^64^Cu-ATSM and ^18^F-FDG in the tumour pieces from tumour 3 (Table 4). For the other tumours the range in SUVs might be too narrow to find a significant correlation. Furthermore the fact that no significant correlations or trends were seen for the correlations between ^64^Cu-ATSM and ^18^F-FDG and the gene expression of the endogenous markers of angiogenesis in tumour 3 may not necessarily be interpreted as a negative result, but rather as an indication that different and further information about the micro regional molecular aspects are gained through simultaneous investigations using several molecular techniques. The gene expression analysis of endogenous markers in the present study thus indicated different molecular expressions in the examined tumours despite their same histopathology. A very recent extensive study of molecular genetic patterns in 12 different human cancer types similarly revealed the existence of subtypes within specific cancer types and additionally gathering of different histopathologic tumour types into the same molecular subtype [73]. This indicates, that in the future malignant tumours should not only be classified according to the traditional histopathologic classification, but also or rather according to a molecular classification. Such a classification may be facilitated by use of molecular imaging techniques as well as endogenous markers.

### Correlations between endogenous markers of proliferation and ^64^Cu-ATSM and ^18^F-FDG

With regard to proliferation as evidenced by Ki-67 protein and gene expression, a discrepancy was seen between the correlations to the two tracers ^64^Cu-ATSM and ^18^F-FDG when using gene expression and IHC, respectively. The results of the gene expression analysis of Ki-67 showed more positive significant correlations for ^18^F-FDG than for ^64^Cu-ATSM (Fig 5), indicating that ^18^F-FDG may be a better marker of proliferation. With Ki-67 IHC no significant correlations to either ^64^Cu-ATSM or ^18^F-FDG could be registered. Possible explanations for this difference in results may be: 1) that tumour pieces used for gene expression analysis and IHC originated from different areas of the tumour; 2) that post transcriptional or translational changes happen between the mRNA measured during qPCR and the protein measured during IHC; 3) that Ki-67 IHC staining was too scarce and homogenous; 4) the limitations associated with comparing Ki-67 IHC and AR images having very different image resolutions. However we tried to avoid small regional discrepancies in uptake and possible manual co-registration inaccuracies by investigating areas of 420 x 420 μm without compromising the interest of getting micro regional information.

Studies in different human cancers have also revealed significant correlations between Ki-67 and ^18^F-FDG e.g. in lymphomas [74], non-small-cell lung cancer [75, 76] and bone and soft tissue sarcomas [77]. However a preliminary study of human malignant melanomas revealed no significant link between ^18^F-FDG uptake and Ki-67 grades [78]. All these studies compared ^18^F-FDG uptake from PET scans with Ki-67 immunostaining in contrast to the present study where the micro regional tracer uptake is correlated to gene expression of Ki-67. However a very recent study in human colon cancer showed a positive correlation between maximum ^18^F-FDG uptake from PET scans and the gene-expression of Ki-67 [79]. Studies comparing ^64^Cu-ATSM and Ki-67 are scarcer and only preclinical comparing immunohistochemistry and autoradiographies [41, 42]. Therefore the significant correlations seen between ^18^F-FDG and Ki-67 gene expression in the present study represent special information about the micro regional relationship between glycolysis and proliferation in solid tumours, which is in agreement with our hypothesis.

## Conclusion

In conclusion this study of canine soft tissue sarcomas revealed that both ^64^Cu-ATSM and ^18^F-FDG uptake is heterogenous across multiple micro regions within and between tumours of the same histopathologic class. At the micro regional level ^64^Cu-ATSM and ^18^F-FDG uptakes and distributions only correlated moderately indicating different biological aspects visualized. ^18^F-FDG better reflected cell proliferation as measured by Ki-67 gene expression than did ^64^Cu-ATSM. Thus each PET tracer contributes with distinct information about the micro regional environment within the cancer. Using endogenous molecular markers also adds relevant information about the microenvironment and heterogeneity and perhaps a molecular taxonomy of cancers may in the future become as important as that of traditional histopathologic classification of cancers. Since the number and types of tumours in the present study were limited, further studies are necessary to investigate whether the observations in the present study are generalizable.

## Supporting Information

S1 Fig Correlations between glycolytic, hypoxic and angiogenic gene expressions and ^64^Cu-ATSM and ^18^F-FDG uptake, tumours 2 and 4. Spearman’s Rank correlations (ρ) and p-values for comparison of gene expressions for GLUT1, GLUT3, HIF-1α, CAIX, VEGFA and TF respectively and tumour uptake of ^18^ F-FDG and ^64^Cu-ATSM calculated as standardized uptake value (SUV) from gamma counts. Rows 1 and 2 (**A)** show data for tumour pieces from canine cancer patient 2, while rows 3 and 4 (**B**) show results for patient 4. n is the number of tumour pieces included in the final analysis. *n*.*s*. not significant. Significant correlations are written in bold.
(TIFF)
