# Supplementary figures and images for: Micro Regional Heterogeneity of 64Cu-ATSM and 18F-FDG Uptake in Canine Soft Tissue Sarcomas: Relation to Cell Proliferation, Hypoxia and Glycolysis
Source: PLoS One. 2015 Oct 26;10(10):e0141379. doi: 10.1371/journal.pone.0141379 (PMC4621038; doi:10.1371/journal.pone.0141379)

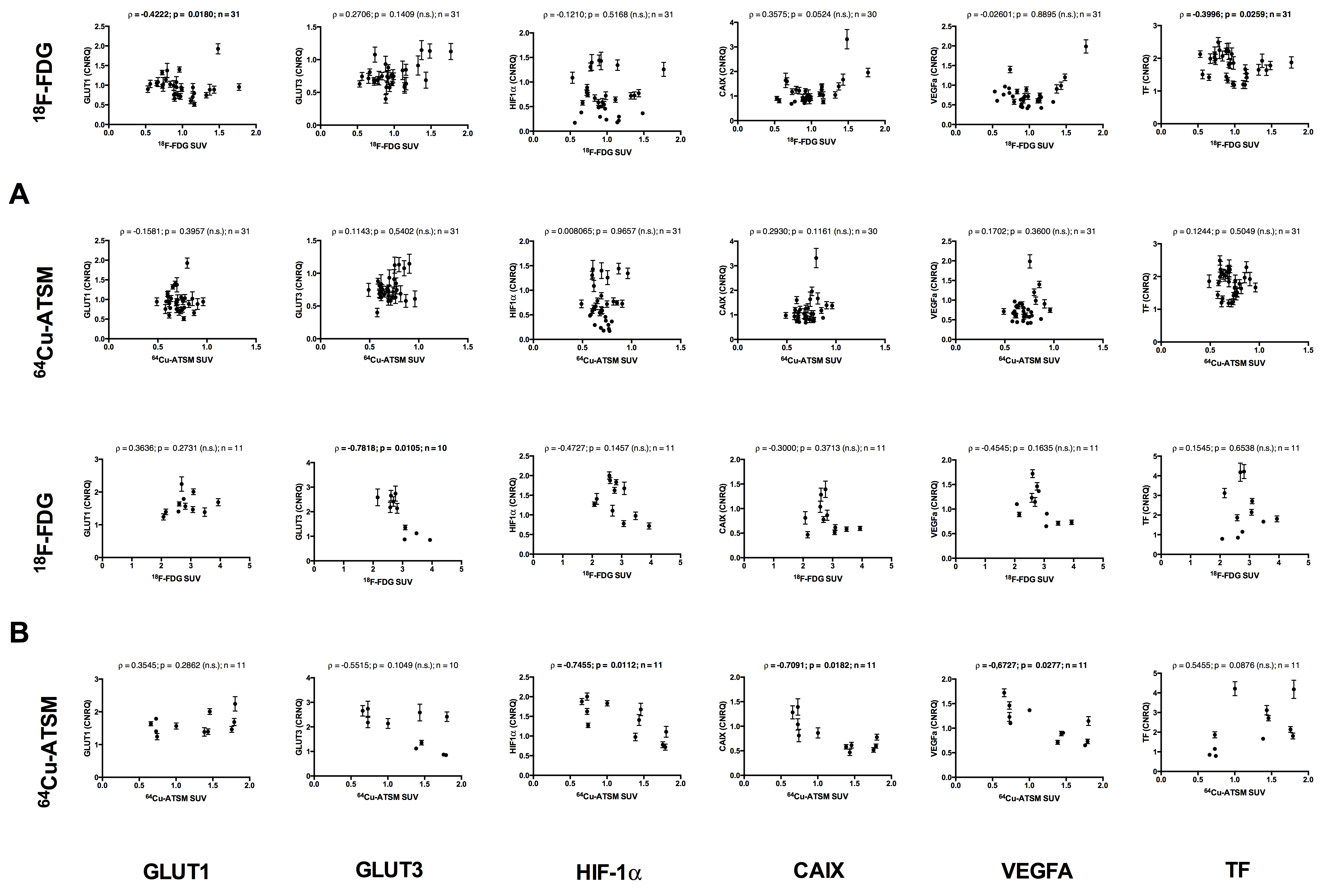

Supplement: S1 Fig — Spearman’s Rank correlations (ρ) and p-values for comparison of gene expressions for GLUT1, GLUT3, HIF-1α, CAIX, VEGFA and TF respectively and tumour uptake of 18 F-FDG and 64Cu-ATSM calculated as standardized uptake value (SUV) from gamma counts. Rows 1 and 2 (A) show data for tumour pieces from canine cancer patient 2, while rows 3 and 4 (B) show results for patient 4. n is the number of tumour pieces included in the final analysis. n.s. not significant. Significant correlations are written in bold. (TIFF) [file pone.0141379.s001.tiff]
